# Increased Mosquito Midgut Infection by Dengue Virus Recruitment of Plasmin Is Blocked by an Endogenous Kazal-type Inhibitor

**DOI:** 10.1016/j.isci.2019.10.056

**Published:** 2019-10-31

**Authors:** Karthik Ramesh, Varsha A. Walvekar, Benjamin Wong, Ahmed Mahmoud Mohammed Sayed, Dorothée Missé, R. Manjunatha Kini, Yu Keung Mok, Julien Pompon

**Affiliations:** 1 Department of Biological Sciences, National University of Singapore, 16 Science Drive 4, Singapore 117558, Singapore; 2 Program in Emerging Infectious Disease, Duke-NUS Medical School, 8 College Road, Singapore 169857, Singapore; 3 Assiut University, Department of Chemistry, Faculty of Science, Assiut 71516, Egypt; 4 MIVEGEC, UMR IRD 224-CNRS5290-Université de Montpellier, Montpellier, France

**Keywords:** Virology, Entomology

## Abstract

Dengue symptoms include alteration of blood coagulation and fibrinolysis, causing severe hemorrhage and death. Here, we demonstrate that higher concentration of plasmin, the human fibrinolytic factor, in blood meal enhances dengue virus (DENV) infection in mosquito midgut and dissemination in mosquitoes. We also show that mosquitoes express a plasmin-selective Kazal-type inhibitor (AaTI) in the midgut to inhibit plasmin proteolysis and revert the enhanced infection. Using bio-layer interferometry, we show that DENV, plasmin, and AaTI interact to form a tripartite complex. Eventually, plasmin increases midgut internalization of dextran molecules and this is reverted by AaTI. Our study demonstrates that (1) DENV recruits plasmin to increase local proteolytic activity in the midgut, thus degrading the glycocalyx and enhancing DENV internalization and (2) AaTI can act as a transmission-blocking agent by inhibiting plasmin proteolysis. Our results indicate that dengue pathogenesis enhances DENV fitness by increasing its infectivity to mosquitoes.

## Highlights

• Higher level of plasmin increases dengue virus internalization in mosquito midgut
• Dengue virus captures plasmin to use its proteolytic activity
• Mosquito Kazal inhibitor inhibits plasmin proteolysis and reduces infection

## Graphical Abstract

**Figure:**
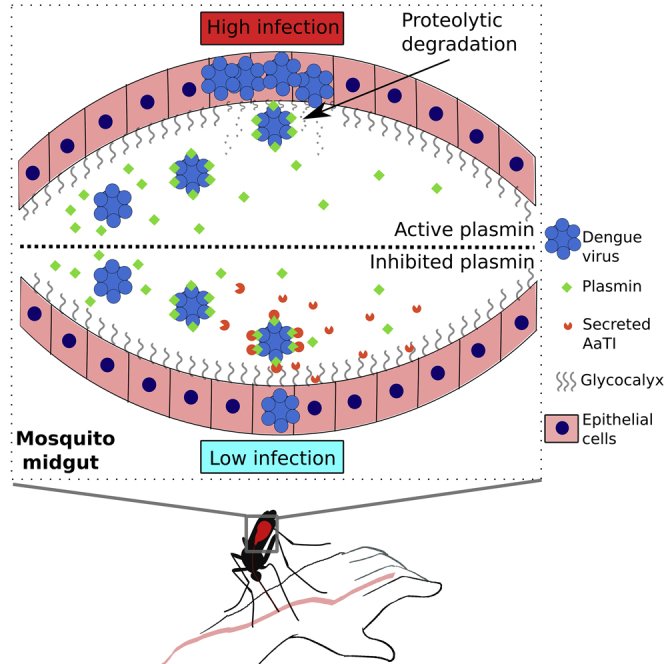

## Introduction

Pathogens alter host physiology in various ways. Vector-borne pathogens often influence three-way interactions involving host, vector, and pathogen (Mauck et al., 2012, Ewald, 1995, Hurd, 2003). Those alterations that maximize transmission success get fixated through natural selection (Ewald, 1995, Anderson and Robert, 1992). Therefore, deciphering how host alterations influence transmission is critical to understand virulence evolution. This knowledge can contribute toward developing strategies to tackle important public health problems caused by vector-borne diseases.

Dengue induces a wide range of symptoms, from none to hemorrhage that can lead to death if not properly managed (Huang et al., 2001, Lee et al., 2017, Guzman and Harris, 2015). The four dengue virus types (DENV1–4) infect approximately 390 million people yearly, causing the most prevalent arboviral disease in the world (Bhatt et al., 2013). DENV cycles between humans and *Aedes aegypti* mosquitoes that acquire and transmit the virus during biting (Westaway, 1987). For successful transmission, DENV ingested from infected humans has to first infect and multiply in the mosquito's midgut epithelial cells. The viruses then disseminate into secondary tissues such as hemocyte and muscles and finally infect the salivary glands, from which they are expectorated in the saliva during subsequent biting (Salazar et al., 2007). However, only a small proportion of ingested DENV initiates midgut infection, creating a barrier that determines mosquito transmission (Franz et al., 2015). Although previous reports have shown that DENV can alter host blood factors (Chuang et al., 2013), little is known about how these factors present in the ingested blood influence midgut infection.

Fibrinolysis is one of the aggravating factors associated with dengue-induced vascular bleeding in children (Sosothikul et al., 2007) and adults (Orsi et al., 2013, Huang et al., 2001). Fibrinolysis is mediated through fibrin clot degradation by the broad-spectrum serine protease plasmin (Cesarman-Maus and Hajjar, 2005). Unchecked plasmin can cause generalized hemorrhagic state within minutes (Ponting et al., 1992). Interestingly, some pathogens recruit circulating plasmin or its zymogen form, plasminogen, to degrade extracellular matrix, thereby facilitating tissue barrier penetration (Lottenberg et al., 1994, Ehinger et al., 2004, Coleman et al., 1997, Sun et al., 2004, Goto et al., 2001). For instance, the parasite *Plasmodium* sp. that causes malaria is transmitted by *Anopheles* mosquitoes and captures plasminogen in the human blood (Ghosh et al., 2011). Subsequent plasminogen activation into plasmin increases mosquito midgut infection by the parasite. However, it is unknown if plasmin stimulates DENV infection. Such knowledge would shed new light on the Cause-and-Effect interaction between pathogenic fibrinolysis, virus infectivity to mosquitoes, and the resulting virus fitness.

In the absence of therapeutics and efficient vaccine against DENV (Barrows et al., 2018, Villar et al., 2015, Sabchareon et al., 2012), transmission-blocking agents represent a promising intervention to curb epidemics. When administered to humans, these agents could increase the barrier to midgut infection. Although *Aedes aegypti* possesses a Kazal-type serine protease inhibitor (hereafter called AaTI) (VectorBase: AAEL006007) that is expressed in the midgut and binds to plasmin, its inhibitory capacity is unknown (Rimphanitchayakit and Tassanakajon, 2010, Watanabe et al., 2010). AaTI contains a single Kazal domain that is structurally constrained by three disulfide bridges to enable stoichiometric binding to proteolytic sites in a lock-and-key manner (Laskowski and Kato, 1980). Similarly to other serine protease inhibitors, invertebrate Kazal-type proteins regulate blood feeding, autophagy, and host-pathogens interactions (Rimphanitchayakit and Tassanakajon, 2010). Because of their specificity and protease inhibition property, serine protease inhibitors have been proposed as therapeutic agents (Masurier et al., 2018).

Here, we investigated how blood changes triggered by dengue pathogenesis influence mosquito infection. We tested whether blood plasmin increases DENV infection in *Ae. aegypti* mosquitoes. We also tested whether midgut-expressed AaTI inhibits plasmin-mediated infection. We discovered that plasmin induces, whereas AaTI limits infection in the midgut lumen. We further determined that DENV particles recruit plasmin, which in turn binds to AaTI to inhibit plasmin proteolysis and revert plasmin infection enhancement. Eventually, we reported that midgut internalization was increased following a blood meal with both DENV and plasmin and that the increase was reverted by AaTI. Collectively, our results reveal how human plasmin and AaTI interaction influences DENV mosquito infection. At the intersection between pathogenesis and vector competence, our study suggests that a human blood component related to dengue symptomatology increases DENV fitness by enhancing mosquito infection. We also identified an associated transmission-blocking candidate.

## Results

### Plasmin Enhances Dengue Virus Infection of Mosquito Midgut

To test whether plasmin increases DENV infectivity, we orally infected female *Ae. aegypti* with pig blood supplemented with human plasmin. We first conducted a preliminary dose-response analysis to determine plasmin effective concentration. Since the blood plasmin levels in healthy humans and patients with dengue are unknown, we tested concentrations around the reported average concentration of plasminogen in healthy human plasma: 2.4 μM (Collen and Lijnen, 1986). We reasoned that plasmin concentration could not exceed that of plasminogen. In control mosquitoes, plasmin solution volume was replaced by RPMI media. Blood inoculum was 107 pfu/mL, which is a biologically relevant titer within the range of viremia measured in hospitalized children during fever onset (Vaughn et al., 2000). At 7 days post oral infection, when DENV has infected midgut and disseminated (Pompon et al., 2017, Salazar et al., 2007), we collected the whole mosquitoes and calculated the titer as plaque-forming units (pfu) per mosquito. The infection level (measured as pfu per infected mosquito) was increased when plasmin concentrations were ≥1.2 μM (Figure S2). Interestingly, infection rate, calculated as the number of mosquitoes with at least 1 pfu over total sampled blood-fed mosquitoes, was not affected by plasmin addition, even for concentrations as high as 6 μM (Figure S2). Because 1.2 μM represents a plasminogen activation rate of 50% and to undertake a more conservative approach by using the lowest effective concentration, we used 1.2 μM of plasmin in subsequent studies.

**Figure 1:**
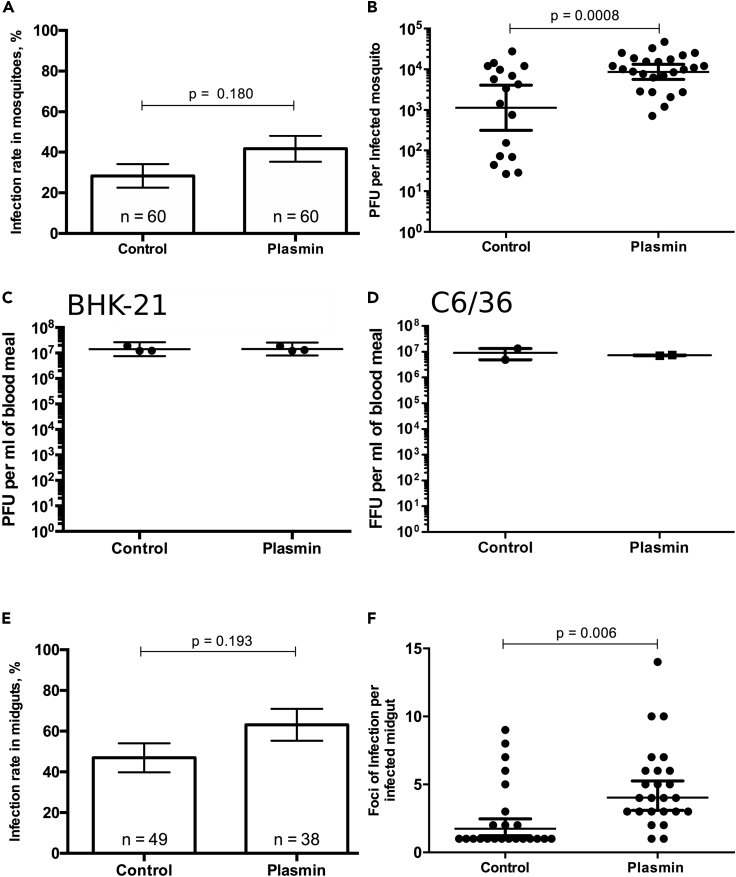
Impact of Plasmin Blood Supplementation on DENV Infection (A–D) (A) Infection rate in mosquitoes and (B) plaque-forming units (pfu) per infected mosquito 7 days post feeding on infectious blood meal supplemented with either plasmin or control. Titers of blood meals supplemented with either plasmin or control as (C) plaque-forming units (pfu) per milliliter with mammalian cells (BHK-21) and (D) focus forming unit (ffu) per milliliter with mosquito cells (C6/36). (E and F) (E) Infection rate in midgut and (F) foci of infection per infected midgut 3 days post feeding on infectious blood meal supplemented with plasmin or control. (A and E) Bars show percentages. Differences were tested with χ^2^ test. n, number of mosquito samples. (B–D) Line represents geometric mean ±95% CI. Differences were tested with t test on log-transformed values. (F) Lines represent arithmetic mean ± SEM. (B–D and F) Each point represents one sample.

Further replication of our experiments showed that plasmin supplementation at 1.2 μM did not alter blood feeding rate or survival of mosquitoes (Figures S3 and S4), both of which are indicative of vector capacity (Kramer and Ciota, 2015). Although the infection rate was not significantly increased with plasmin addition (Figure 1A), DENV titer per infected mosquito was significantly (p value = 0.0008) increased by 2.24-fold (Figure 1B). To test whether plasmin also modified DENV infectivity in mammalian and mosquito cells, we titered the artificial blood meals used for oral infection in hamster kidney (BHK-21) and *Aedes albopictus* cells (C6/36). As expected from the inoculum dilution, titers in these cells were approximately 10^7^ pfu/mL and were not altered by plasmin addition (Figures 1C and 1D). These suggest that plasmin increase of infection is specific to the midgut environment.

We hypothesized that plasmin-mediated increase in mosquito titers was caused by enhanced initiation of midgut infection. To test this, we orally infected mosquitoes with blood supplemented with 1.2 μM of plasmin and immunostained the midguts for DENV envelope at 3 days post infection, when DENV has multiplied in midgut cells and formed foci of infection (Salazar et al., 2007). We reasoned that one focus of infection resulted from one infected cell (Figure S5). Control mosquitoes were fed with infectious blood without plasmin. Similar to the whole mosquitoes, plasmin did not increase midgut infection rate (p value = 0.193; Figure 1E), as defined by the percentage of blood-fed midguts with at least one focus, but significantly (p value = 0.006) increased the number of infection foci per infected midgut from 2.52 to 4.84 (Figure 1F). Initiation of midgut infection by an average of two virions was previously reported for Venezuelan Equine Encephalitis Virus (Alphavirus) in *Culex* mosquito vectors (Forrester et al., 2012). Taken together, our results demonstrate that blood plasmin enhances DENV infection onset in the midgut, resulting in higher infection rate and viral dissemination in the whole mosquitoes.

### A Kazal Inhibitor of Plasmin Limits Infection in Midgut Lumen

**Figure 2:**
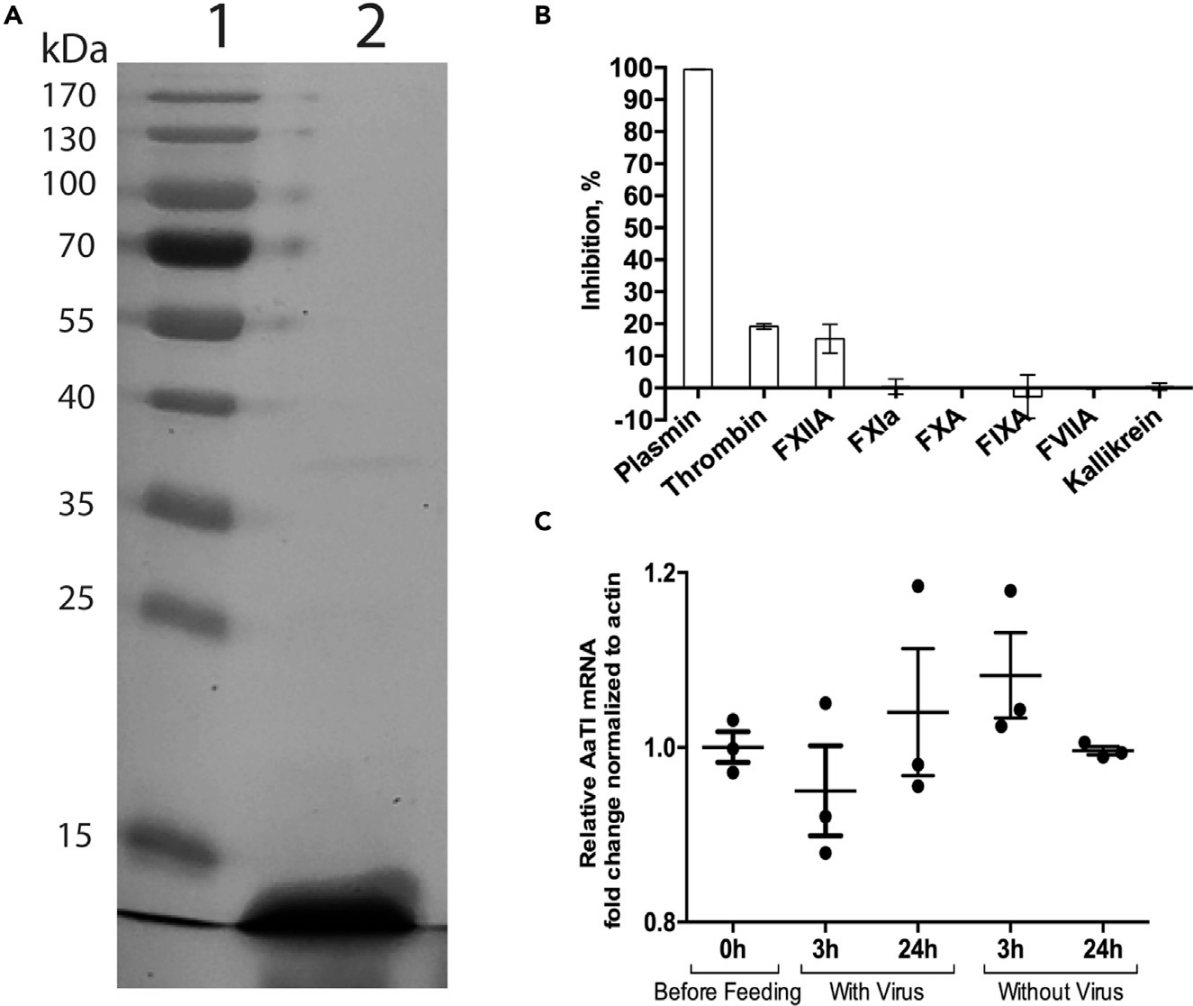
Characterization of AaTI Inhibition and Expression in Midgut (A) SDS-PAGE gel of expressed rAaTI. Lane1, Protein ladder; Lane2, Eluate. (B) rAaTI inhibitory activity against serine proteases involved in blood coagulation. (C) AaTI mRNA expression in midgut before and at 3 and 24 h post feeding on infectious or non-infectious blood meals. Lines represent mean ± SEM. Each point represents a sample containing 10 midguts.

Previous studies have shown that AaTI, a Kazal inhibitor, is expressed in midgut and binds to plasmin (Watanabe et al., 2010, Watanabe et al., 2011). Based on these findings, we hypothesized that AaTI interferes with the plasmin-mediated infection. To test this, we expressed recombinant AaTI (rAaTI) and characterized its protease specificity *in vitro* and its midgut expression profile. The rAaTI was expressed in a bacterial system and purified to obtain a 9.85 kDa protein (Figure 2A) inclusive of His-tag. Using an *in vitro* chromogenic assay, we screened the inhibitory profile of rAaTI (30 μM) against 10 proteases involved in blood coagulation and observed 100% inhibition of plasmin proteolytic activity (Figure 2B). However, the same concentration of rAaTI only marginally inhibited thrombin and FXIIA activities and had no inhibitory activity on other serine proteases. Next, we performed curve inhibition assay and calculated that plasmin's IC_50_ for rAaTI inhibition was 118 nM (Figure S6). Additionally, we quantified *AaTI* mRNA in the midgut before blood-feeding and at 3 and 24 h post oral feeding on non-infectious or infectious blood meals. *AaTI* midgut expression was not affected by blood-feeding or infection (Figure 2C). These results showed that AaTI is constitutively expressed in the midgut, where our enzymatic assay indicated that it can inhibit the proteolytic activity of plasmin.

**Figure 3:**
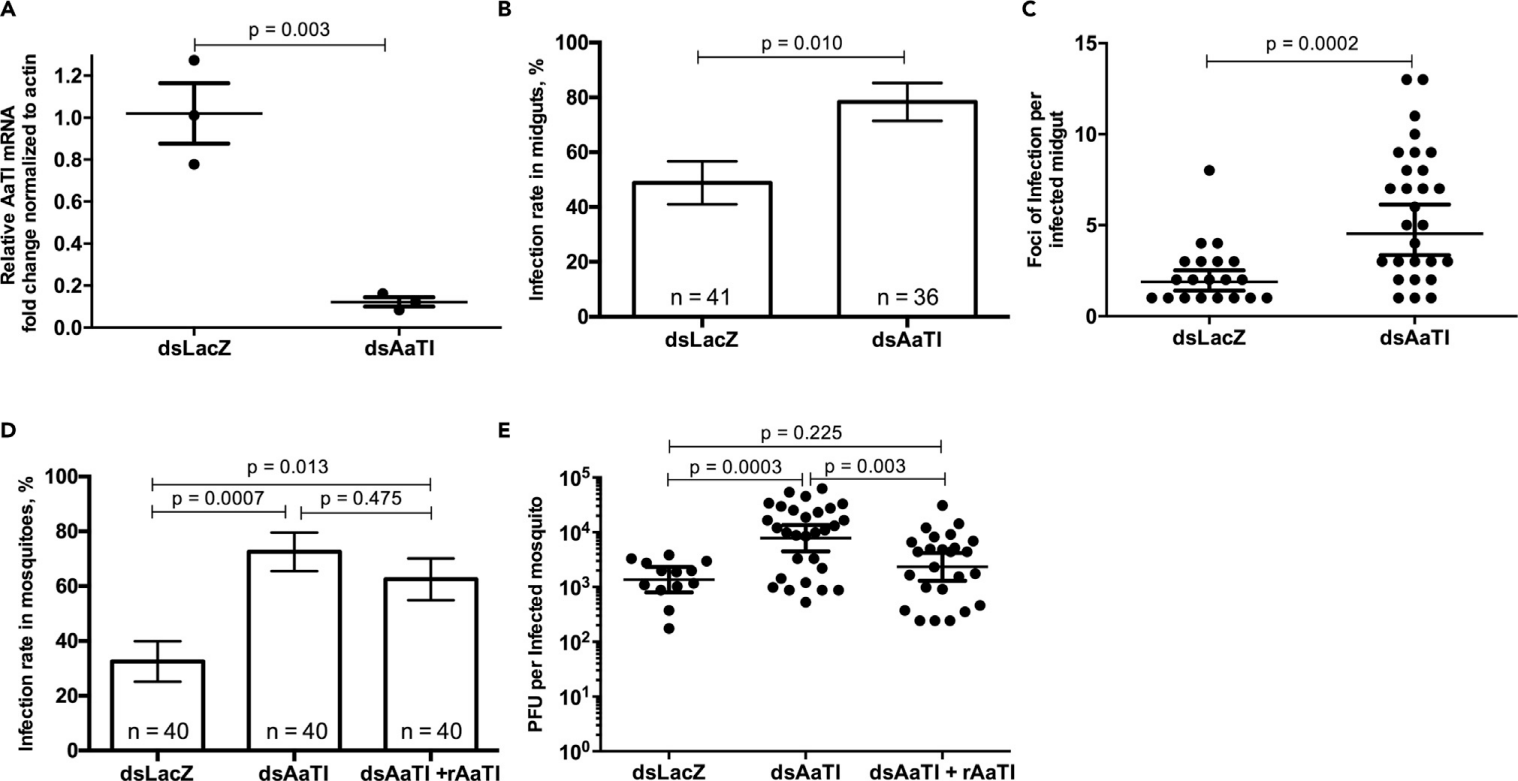
Impact of AaTI on DENV Infection (A) *AaTI* mRNA level after RNAi depletion and oral infection. Mosquitoes were injected with dsRNA against *AaTI* (dsAaTI) or *LacZ* (dsLacZ) 4 days before oral infection. *AaTI* mRNA was quantified in midguts at the time of quantification of midgut infection and normalized to *actin* mRNA level. Each point represents one sample containing 10 midguts. (B and C) (B) Infection rate in the midguts and (C) foci of infection per infected midgut of dsAaTI- and dsLacZ-injected mosquitoes 3 days post oral infection. (D and E) (D) Infection rate in mosquitoes and (E) pfu per infected dsAaTI- or dsLacZ-injected mosquito 7 days post feeding on blood with or without rAaTI. (A, C, and E) Each point represents one sample. Differences were tested with t test. (A and C) Lines show means ± SEM. (B and D) Bars show percentage ± SE. Differences were tested with χ^2^ test. n, number of mosquito samples. (E) Differences were tested with t test after log-transformation. Lines show geometric means ± 95% CI.

To determine if AaTI modulates midgut infection, we depleted AaTI by injecting dsRNA against *AaTI* (dsAaTI). dsRNA against the *LacZ* (dsLacZ) bacterial gene was injected as a control. On the fifth day after dsRNA injection, mosquitoes were orally fed with infectious blood meal with 10^7^ pfu/mL of DENV and human serum, which naturally contains plasmin. This blood meal was not supplemented with pure plasmin as earlier. Using a standard curve, we calculated that plasmin concentration in the blood meal was 19.2 ± 0.44 nM (Figure S7). At 3 days post infection, we validated *AaTI* mRNA depletion in the midguts (Figure 3A) and dissected the tissues for DENV envelope immunostaining and foci count. AaTI depletion significantly increased both the infection rate from 48.7 to 77.7% (p value = 0.010; Figure 3B) and the number of infection foci per infected midgut from 2.3 to 5.78 (p value = 0.0002; Figure 3C). Together, these results revealed that AaTI inhibits DENV midgut infection.

Because AaTI contains a signal peptide that may facilitate its secretion, we tested whether AaTI antiviral function takes place in the midgut lumen using a rescue experiment. dsAaTI-injected mosquitoes were fed with infectious blood meal complemented with 100 μM of rAaTI protein. AaTI was mostly present in the lumen as dsAaTI depleted AaTI from all tissues, including the midguts (Figure 3A). The infectious blood meal also contained 19.2 nM of plasmin from the human serum (Figure S7), resulting in ∼1:5,000 ratio of plasmin:rAaTI. We chose this ratio to maximize proteolysis inhibition and reveal rAaTI function in midgut infection. We added two controls: (1) dsLacZ- and (2) dsAaTI-injected mosquitoes fed on infectious blood without rAaTI. At 7 days post oral infection, we titered the whole mosquitoes. As shown for midguts (Figures 3B and 3C), AaTI depletion significantly increased infection rate in mosquitoes (p value = 0.0007; Figure 3D) and titer per infected mosquito (p value = 0.0003; Figure 3E) when compared with dsLacZ control. Interestingly, rAaTI complementation significantly (p value = 0.003) diminished the infection level in AaTI-depleted mosquitoes, restoring it to that measured in dsLacZ control (Figure 3E). However, rAaTI complementation only moderately (p value = 0.475) reduced the infection rate (Figure 3D), suggesting that rAaTI blood complementation was not sufficient to rescue AaTI depletion in the lumen and epithelial cells. Taken together, the results suggest that AaTI inhibits DENV infection in the midgut lumen.

### AaTI Blocks Plasmin-Mediated Infection

**Figure 4:**
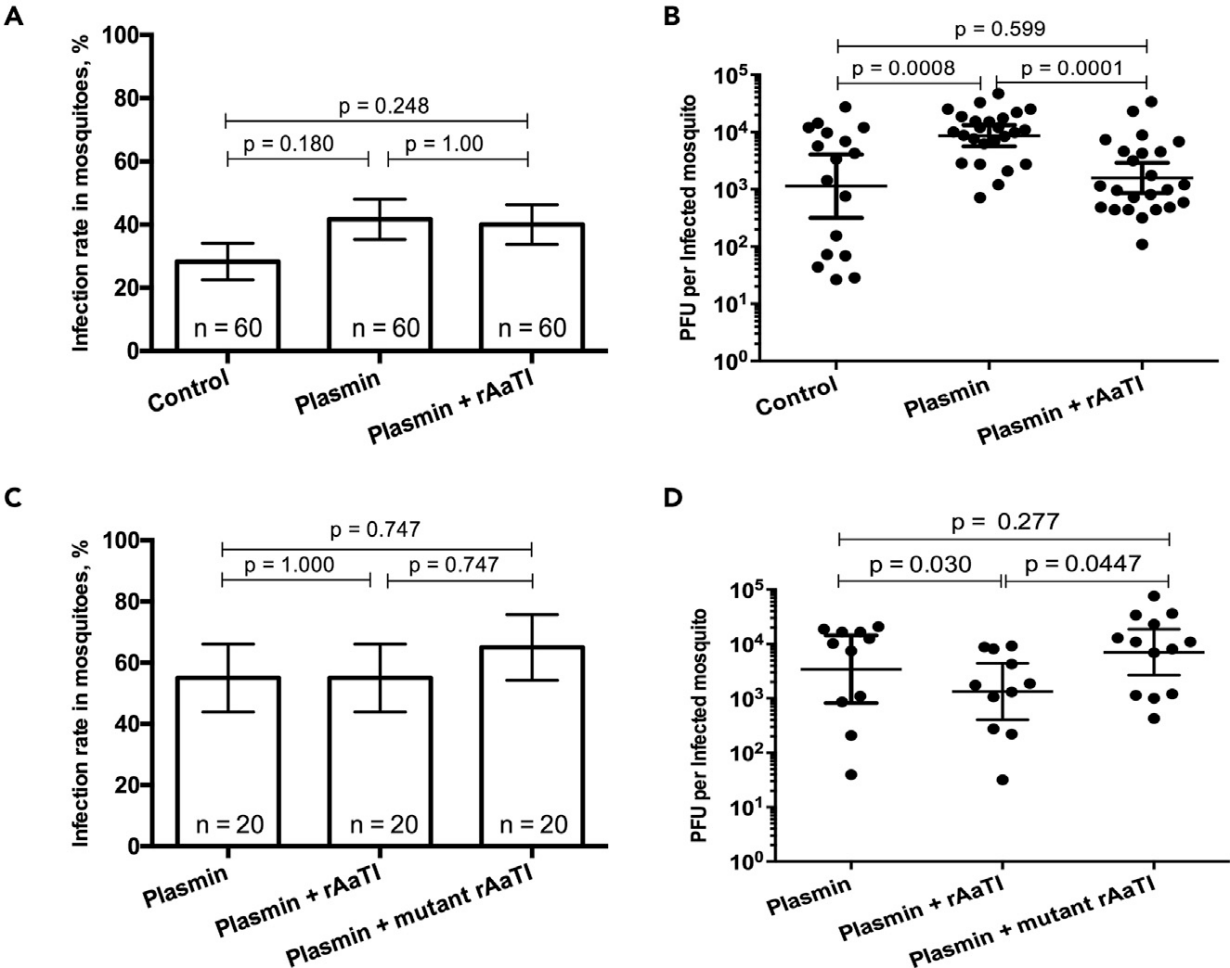
Impact of Plasmin-AaTI Interaction on DENV (A and B) (A) Infection rate in mosquitoes and (B) pfu per infected mosquito 7 days post feeding on infectious blood meal supplemented with plasmin, plasmin and rAaTI, or control. (C and D) (C) Infection rate in mosquitoes and (D) pfu per infected mosquito 7 days post feeding on an infectious blood meal supplemented with plasmin, plasmin and rAaTI, or plasmin and mutant rAaTI. (A and C) Bars show percentages ± SE. Differences were tested with χ^2^ test. n, number of mosquito samples. (B and D) Each point represents one mosquito sample. Lines show geometric means ± 95% CI. Differences were tested with t test after log-transformation.

We next tested whether there is a functional link among plasmin and AaTI pro- and anti-viral functions. To determine whether AaTI can reduce plasmin-mediated DENV infection, we orally infected mosquitoes with infectious blood supplemented with 1.2 μM plasmin and 100 μM rAaTI, resulting in a plasmin:rAaTI ratio of ∼1.2:100. As controls, we used blood without plasmin and rAaTI supplementations and blood with plasmin. We titered whole mosquitoes at 7 days post infection. Although the infection rate did not vary (Figure 4A), rAaTI addition to plasmin-supplemented blood significantly (p value = 0.0001) decreased the titer when compared with plasmin only blood, restoring it to the level measured in no-plasmin no-rAaTI control mosquitoes (Figure 4B). Although we did not test for AaTI inhibition of other unknown proteases and our results do not establish a causal relationship between the two molecules, they suggest that AaTI can antagonize plasmin-mediated function.

To identify a functional link between AaTI and plasmin, we generated a mutant rAaTI that did not inhibit plasmin activity by substituting the P1 residue from R to A. The P1 position in AaTI-type inhibitors regulates the serine protease inhibitor function (Laskowski and Kato, 1980). The mutant rAaTI was expressed in a bacterial system, purified, and tested for its inhibitory activity against plasmin proteolysis using the chromogenic assay. As expected, the mutant rAaTI did not inhibit human plasmin when compared with wild-type rAaTI (Figure S6). We then orally infected mosquitoes with blood supplemented with either wild-type or mutant rAaTI (100 μM) or control. All blood meals also contained 1.2 μM of plasmin to induce DENV infection. At 7 days post infection, the infection rate was not altered by any conditions (Figure 4C). However, the wild-type rAaTI reduced plasmin-mediated increase in titer per infected mosquito, whereas the mutant rAaTI failed to do so (Figure 4D). This indicates that AaTI inhibition of plasmin proteolysis reduces plasmin-mediated infection. Together, our results strongly suggest that DENV infectivity in midguts depends on the balance between AaTI and plasmin.

### DENV Particles Recruit Plasmin that Can Be Bound by AaTI

**Figure 5:**
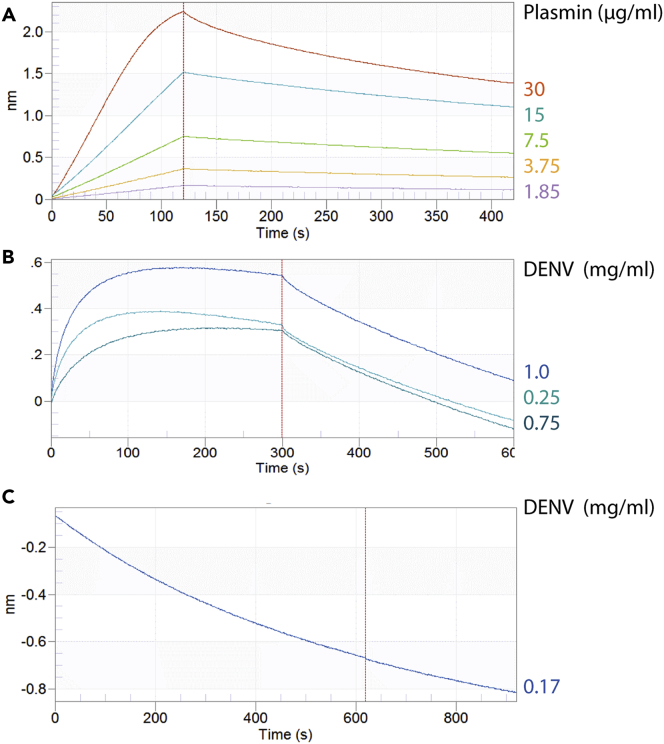
Physical Interaction between Plasmin, AaTI, and DENV (A) Association and dissociation curves of different plasmin concentrations to AaTI preloaded biosensors. (B) Association and dissociation curves of different DENV concentrations to plasmin-AaTI complex. (C) Association and dissociation curves for DENV to plasmin-trypsin complex. The red dotted lines separate association (left) and dissociation (right). The curves were used to compute equilibrium dissociation constant by globally fitting the rate equation for 1:1 kinetics.

DENV envelope protein binds plasminogen, the zymogen of plasmin (Monroy and Ruiz, 2000, Huang et al., 2001), whereas AaTI binds plasmin (Rimphanitchayakit and Tassanakajon, 2010, Watanabe et al., 2010). To characterize the physical interactions among DENV, plasmin, and AaTI, we used Bio-Layer Interferometry with sequential binding assays. First, we quantified plasmin-rAaTI interaction by immersing the rAaTI-preloaded biosensors into plasmin solutions. Plasmin efficiently bound to rAaTI (Figures 5A and S8A) with a K_d_ of 62.8 nM (R^2^ = 0.99). Next, to test whether AaTI interferes with DENV-plasmin interaction, the biosensors preloaded with complexed rAaTI-plasmin were immersed in solutions containing different DENV concentrations. To control for AaTI-DENV interaction, which was minimal (Figure S8B), values for DENV interaction with rAaTI were used for baseline correction. DENV exhibited a dose-dependent interaction with plasmin and a K_d_ of 3.36 μM (R^2^ = 0.93) (Figure 5B). These results demonstrate a strong interaction between DENV and plasmin, which can concomitantly bind to AaTI.

To estimate the specificity of DENV interaction with proteases, we tested whether DENV particles interact with trypsin, another known inhibitory target for AaTI (Watanabe et al., 2010). Trypsin is secreted in mosquito midgut and influences DENV infection by altering blood digestion (Molina-Cruz et al., 2005). Ni-NTA biosensors were loaded with rAaTI and immersed in a trypsin solution to form rAaTI-trypsin complex (Figure S8C). The rAaTI-trypsin preloaded biosensors were then plunged into DENV solution. In contrast to plasmin, DENV did not bind the rAaTI-trypsin complex (Figures 5C and S8C). Together, the physical interactions among DENV, plasmin, and AaTI suggest that DENV specifically recruits plasmin to enhance infection onset in mosquito midgut through plasmin proteolysis, which is inhibited by AaTI binding.

### Plasmin Supplementation to Infectious Blood Increases Midgut Internalization, which Is Reverted by AaTI

**Figure 6:**
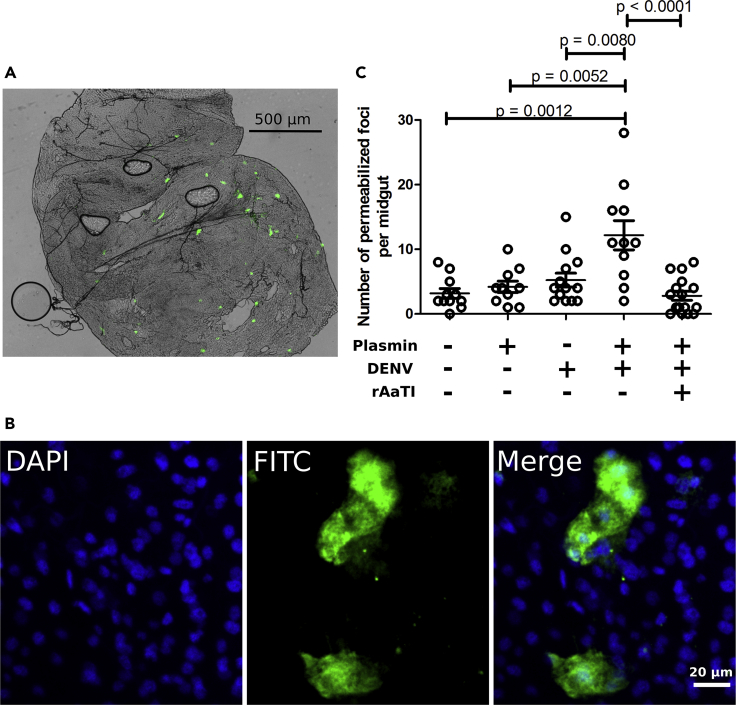
Midgut Internalization by Plasmin, DENV, and rAaTI (A) Representation of midgut tissue with dextran foci from mosquitoes fed on FITC-dextran-containing blood supplemented with plasmin and DENV. Green areas represent FITC-dextran penetration inside the midgut cells. (B) Zoom on dextran foci with a confocal microscope. DAPI staining indicates nucleus. FITC indicates dextran molecules. (C) Number of permeabilized foci per midgut when blood meals were supplemented with (1) plasmin, (2) DENV, (3) plasmin and DENV, and (4) plasmin, DENV, and rAaTI. Control midguts were collected after feeding on blood without plasmin, DENV, and rAaTI supplementations. Midguts were dissected at 18 h post oral feeding. Each point represents one midgut. Bars indicate arithmetic means ± SEM. Differences were tested with t test.

To test whether DENV-recruited plasmin proteolysis increases midgut internalization, we used FITC-labeled dextran molecules that are internalized through the same clathrin-dependent endocytosis as DENV (Li et al., 2015, Edwards and Jacobs-Lorena, 2000). Mosquitoes were fed a blood meal with FITC-dextran containing (1) 1.2 μM plasmin, (2) 10^7^ pfu/mL of DENV, (3) 1.2 μM of plasmin and DENV, (4) 1.2 μM of plasmin, DENV, and 100 μM of rAaTI, or (5) control blood meal without added plasmin, DENV, and rAaTI. At 18 h post feeding, we dissected midguts and, interestingly, observed foci of dextran molecules (Figure 6A). This indicates that dextran molecules are efficiently internalized. We further characterized the distribution of dextran molecules within the midgut cells using confocal microscopy and found they were present inside the cytoplasm (Figure 6B), confirming the use of the assay to quantify midgut internalization.

We then counted dextran foci per midgut under the different conditions. When compared with control blood, plasmin and DENV alone did not increase the number of permeabilized foci (Figure 6C). However, supplementation of both plasmin and DENV significantly (p = 0.008) increased the number of dextran foci. This increase was reverted when rAaTI, the plasmin proteolysis inhibitor, was added (Figure 6C). Together, our results suggest that DENV recruitment of plasmin proteolysis increases midgut internalization.

## Discussion

Understanding how human blood factors influence transmission to mosquitoes provides the underlying mechanisms between blood-related pathogenesis and DENV fitness. This can explain epidemiological and virulence observations. In this study, we discovered that DENV recruits plasmin, the human fibrinolysis factor, to increase midgut internalization and enhance the infection onset in mosquito. These results suggest a model whereby DENV exploits human blood plasmin to degrade the glycocalyx (Becker et al., 2015), allowing access to epithelial cells for internalization. Additionally, we demonstrated that a mosquito Kazal-type plasmin inhibitor, AaTI (Watanabe et al., 2010), binds plasmin to antagonize its proviral function by inhibiting its proteolytic activity in the midgut lumen. Our study sparks off two novel hypotheses. First, DENV strains that induce fibrinolytic factors improve DENV fitness and should be under positive selection, partially shaping virulence evolution. Second, the plasmin inhibitor AaTI is a candidate for a transmission-blocking strategy, with potentially higher impact on hemorrhagic patients. Nonetheless, since the impact of plasmin or AaTI additions on mosquito infection is moderate, these hypotheses should be further tested. That DENV remains able to infect midgut in the absence of plasmin excess or with excess of the AaTI plasmin inhibitor also indicates that additional factors shape mosquito infection.

Using Bio-Layer Interferometry, we showed that DENV particles recruit plasmin. Plasmin comprises one serine protease active site and five kringle domains, four of which bind to lysine residues (Law et al., 2013). DENV has been reported to interact with the lysine-binding domain 1 from kringle domains 1–3 of plasminogen, the plasmin zymogen (Monroy and Ruiz, 2000). Since the lysine-binding domain 1 is present in both inactive plasminogen and active plasmin, it is tempting to speculate that DENV particles recruit both plasmin and plasminogen from human blood through the same kringle domain interaction. Conformational changes associated with DENV binding could activate plasminogen into plasmin, which could then be released in blood through binding competition with plasminogen. In addition, DENV particles harbor 80 envelope proteins on their surface (Kuhn et al., 2002, Zhang et al., 2013), offering as many potential plasmin-binding sites. Interestingly, the predicted envelope residues that interact with the lysine-binding domain 1 are highly conserved among flaviviruses (Figure S9), suggesting that plasmin recruitment and its pro-viral function is conserved. Further studies should test whether plasmin increases midgut internalization for ZIKV in *Aedes* spp. and west Nile virus in *Culex* spp.

Plasmin has a broad-spectrum proteolytic activity that can degrade different extracellular matrix (Law et al., 2013, Ploplis and Castellino, 2000), including glycocalyx (Becker et al., 2015). In our studies, plasmin was associated with increased DENV infection in the midgut. We observed that AaTI inhibition on plasmin proteolysis reduces infection, whereas mutant AaTI with loss of inhibitory capacity does not. These imply that plasmin proteolysis is involved in DENV infection. Following blood ingestion, mosquitoes generate a chitin-based extracellular peritrophic matrix “encapsulating” the blood meal to prevent free heme damage (Devenport et al., 2006) and regulate digestion (Villalon et al., 2003). Peritrophic matrix can alter infection by pathogens (Shao et al., 2001), but it does not reduce DENV infection in *Ae*. *aegypti* (Kato et al., 2008). Swift DENV infection in midgut epithelial cells may occur before peritrophic matrix maturation that occurs at 12 h post blood ingestion. Conversely, *Plasmodium* ookinete invasion of midgut cells occurs between 12 and 24 h post blood ingestion and is influenced by peritrophic matrix formation in *Ae*. *aegypti* (Perrone and Spielman, 1988). Past the peritrophic matrix, the luminal side of epithelial cells is covered with the glycocalyx (Lehane et al., 1996), composed of oligosaccharide chains integrated with glycoproteins, proteoglycans, and glycolipids. The glycocalyx is supposed to protect against digestion-related damages. Based on our findings and the literature, we propose that DENV-recruited plasmin degrades the glycocalyx, thereby allowing access to epithelial cells and enhancing internalization and infection. To estimate internalization, we quantified intracellular dextran molecules, which are endocytosed through the same clathrin-dependent pathway as DENV (Li et al., 2015). Dextran internalization requires DENV recruitment of plasmin to degrade the glycocalyx, suggesting that dextran-labeled cells have been made accessible by the presence of DENV. It is, thus, highly likely that the dextran-labelled cells are also infected with DENV. Virus internalization requires interaction with the epithelial cells via different types of membrane receptors (Cruz-Oliveira et al., 2015). Interestingly, mosquito internalization receptors include a 67 kDa enolase that also binds plasmin and plasminogen (Muñoz Mde et al., 2013). Thus, plasmin could play a secondary function in cell internalization.

By demonstrating that blood plasmin promotes mosquito midgut infection, our study revealed a mechanistic link between the dengue-induced fibrinolytic factor and DENV fitness (Conway et al., 2014, Guzman and Harris, 2015). Strikingly, we observed that plasmin addition in the blood boosted midgut infection and virus dissemination (titer in whole mosquitoes), augmenting transmission capacity and, consequently, virus fitness. DENV mechanisms that stimulate fibrinolysis should, thus, be under positive selective pressure. Antibodies against the DENV envelope or NS1 proteins can activate plasminogen through antibody cross-reactivity (Chuang et al., 2011, Chuang et al., 2016, Markoff et al., 1991, Monroy and Ruiz, 2000). Accordingly, plasminogen-reactive antibodies were found in patients with dengue (Chungue et al., 1994, Markoff et al., 1991). Furthermore, envelope proteins from viruses isolated from patients with severe dengue were more efficient at activating plasminogen than the envelope proteins isolated from patients with non-severe dengue (Monroy and Ruiz, 2000). Our results imply that mechanisms of plasminogen activation, hence fibrinolysis, should be under positive selective pressure to improve DENV infection in mosquitoes.

Implications on the positive association between fibrinolysis and virus fitness on disease severity are complex. The clinical course of dengue consists of (1) the febrile phase (3–7 days), when patients can experience sudden onset of fever and flu-like symptoms; (2) the critical phase (2–3 days) at the time of defervescence, when patients can experience severe symptoms such as hemorrhage and shock; and (3) the recovery phase that lasts 2–5 days (Yacoub et al., 2014). Disease severity is determined by the complex interaction between host immunological status and viral genetics (OhAinle et al., 2011). Although infection confers life-long immunity against the infecting serotype, secondary heterologous infection is enhanced by pre-existing cross-reactive non-neutralizing antibodies. These facilitate DENV entry into Fc-γ receptor-bearing cells, a process known as antibody-dependent enhancement (ADE) that results in more severe symptoms (Halstead, 1988). On the other hand, epidemiological studies repeatedly reported that clade replacements within one serotype (therefore not involving ADE) are associated with increased disease severity (OhAinle et al., 2011, Rico-Hesse et al., 1997, Messer et al., 2002, Bennett et al., 2006), indicating that virus genetics influence both severity and fitness. The most compelling example of clade replacement occurred in Latin America where the aggressive Asian DENV-2 genotype replaced the less severe American genotype (Rico-Hesse et al., 1997).

Physiologically, disease severity results from complex interactions, not fully characterized (Yacoub et al., 2013), among viral proteins, vasoactive cytokines, capillary leakage of anticoagulant proteins, endothelial release of procoagulant factors, circulation of heparin-like anticoagulants and activation of plasminogen (Wills et al., 2002, Sosothikul et al., 2007, Monroy and Ruiz, 2000). In the following text, we refer to studies with DENV-infected human subjects to discuss association between disease pathogenesis and DENV transmission. Mosquitoes can be infected from 2 days before and up to 5 days after symptom onset (Nguyen et al., 2013, Nishiura and Halstead, 2007). This corresponds with a window that mostly overlaps with the febrile phase and rarely encompasses symptomatic vascular leakage. Additionally, blood infectiousness to mosquitoes is strongly determined by viremia, which also peaks during the febrile phase (Nguyen et al., 2013). Nonetheless, early or critical phase fibrinolysis could compensate for loss of infectiousness resulting from viremia decrease and extend the transmission time window, thereby increasing DENV fitness. Alternatively, fibrinolysis localized in dermis, where mosquitoes collect blood, could influence infectiousness and explain the higher mosquito infectivity observed in direct feeding on patients with dengue as compared with indirect feeding on intravenously collected blood from the same patients (Duong et al., 2015). This hypothesis is supported by studies using monkeys, where DENV replicates in the skin up to 3 days post viremia termination (Marchette et al., 1973). Taken together, our studies and others suggest that quantification of plasminogen and plasmin concentrations across the clinical course of dengue infection can improve our understanding of the complex interactions among fibrinolysis, disease severity, and transmission.

Currently, there is no cure for dengue (Barrows et al., 2018). The only available vaccine, DENGVAXIA, has variable and poor efficacy against the different serotypes and is not recommended to protect against primo infection. In addition, this vaccine is not licensed for young and old patients, who are the most vulnerable to dengue (Villar et al., 2015, Sabchareon et al., 2012, Capeding et al., 2014). Thus, alternative treatment strategies are required to curb the rise of dengue virus infection (Bhatt et al., 2013). Targeting transmission to vectors is a promising strategy when it aimed at the infection initiation in the mosquito midgut (Londono-Renteria et al., 2016). For instance, immunization against a mosquito C-type lectin that facilitates DENV-2 midgut invasion in *Ae*. *aegypti* significantly reduced transmission (Liu et al., 2014). However, the efficiency of transmission-blocking vaccination is constrained by high antibody titer required at the biting site (Kaslow, 1997). Here, we showed that AaTI, a mosquito Kazal-type serine protease inhibitor, reduced DENV infection when it is added to blood, potentially blocking transmission. In addition, AaTI administration to patients could limit fibrinolysis vascular leakage, although further studies are needed to test its safety and efficacy. Through its dual regulation of symptoms and mosquito infection, AaTI represents an interesting candidate for intervention against DENV.

In conclusion, we demonstrate that dengue-triggered blood alteration increases DENV transmission to mosquitoes, suggesting a mosquito-centered evolutionary pressure for dengue pathogenesis in human. Furthermore, we identified a mosquito inhibitor of this pathogenesis-mediated mechanism of transmission.

### Limitations of the Study

To test the impact of plasmin, we used an oral infection model that combines pig erythrocytes with human plasmin. Although we chose a plasmin concentration that is physiologically relevant to the concentration of plasminogen, this model represents an approximation as there are no data on plasmin increase upon dengue. To remedy this, we measured the concentration of plasmin in the serum of five patients with dengue and two other control humans (data not shown). However, we did not observe significant variations between the two conditions. Several reasons may account for this result: (1) plasmin level may vary between individuals and the appropriate control should have been the same individual before infection; (2) plasminogen activation and thus plasmin concentration is tightly regulated and should vary widely within the body. We used serums collected intravenously that may not represent dengue effect on plasmin level; (3) plasmin concentration upon DENV infection is dynamic and would require sampling throughout the infection cycle. We only had one time point. Testing the dynamics of plasmin concentration throughout the course of dengue would complement our findings and could be used to confirm plasmin pro-viral function using blood of a patient with dengue.

## Methods

All methods can be found in the accompanying Transparent Methods supplemental file.

## Supplemental Information

Document S1. Transparent Methods, Figures S1–S9, and Tables S1 and S2
