# Supplementary material for: Increased Mosquito Midgut Infection by Dengue Virus Recruitment of Plasmin Is Blocked by an Endogenous Kazal-type Inhibitor
Source: iScience. 2019 Oct 31;21:564–76. doi: 10.1016/j.isci.2019.10.056 (PMC6854080; doi:10.1016/j.isci.2019.10.056)
Supplement: Document S1. Transparent Methods, Figures S1–S9, and Tables S1 and S2 [file mmc1.pdf]

ISCI, Volume *21*

Supplemental Information

Increased Mosquito Midgut Infection by Dengue Virus Recruitment of Plasmin Is Blocked by an Endogenous Kazal-type Inhibitor

Karthik Ramesh, Varsha A. Walvekar, Benjamin Wong, Ahmed Mahmoud Mohammed Sayed, Dorothée Missé, R. Manjunatha Kini, Yu Keung Mok, and Julien Pompon

Supplementary Information

A

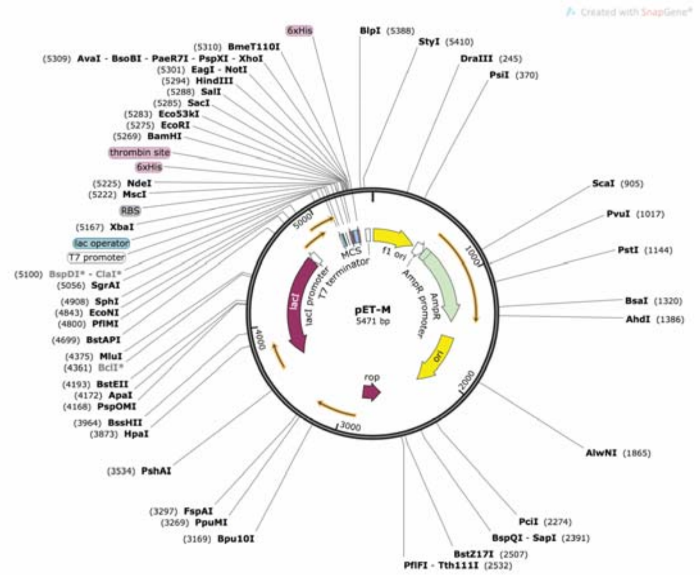

B

ATGCACCATCATCATCATCATTCTTCTGGTCTGGAAGTTCTGTTCCAGGGGCCCCGTAGCGATGCGGAACGTGGCGTGTGCGCATGTCCGCGTA
TTTATATGCCGGTTTGCGGCAGCAACCTGAAAACCTACAACAATGATTGCCTGCTGCGCTGTGAAATTAATTCTGACCTGGGTCGTGCGAACAA
TCTGCGCAAAATCGCGGATCAGGCCTGTGACAACCTGACGGATAACGTCAATGACTTTATCCCGCAAGAATATTAAGAATTC

C

MHHHHHHSSGLEVLFQGPRSDAERGVCACPRIYMPVCGSNLKTYNNDCLLRCEINSDLGRANNLRKIADQACDNLTDNVNDFIPQEY

D

MHHHHHHSSGLEVLFQGPRSDAERGVCACPAIYMPVCGSNLKTYNNDCLLRCEINSDLGRANNLRKIADQACDNLTDNVNDFIPQEY

Figure S1. Description of the construct used for rAaTI expression, Related to Figure 2. (A) Pet-M plasmid map used to clone and express rAaTI with SHuffle T7 bacteria. (B) Bacteria-codon optimized *rAaTI* sequence. (C) rAaTI protein sequence including N-terminal his-tag (blue), precision protease cleavage site (green) and the P1 position residue R31 (red). (D) Mutant rAaTI protein sequence with P1 residue mutated (red).

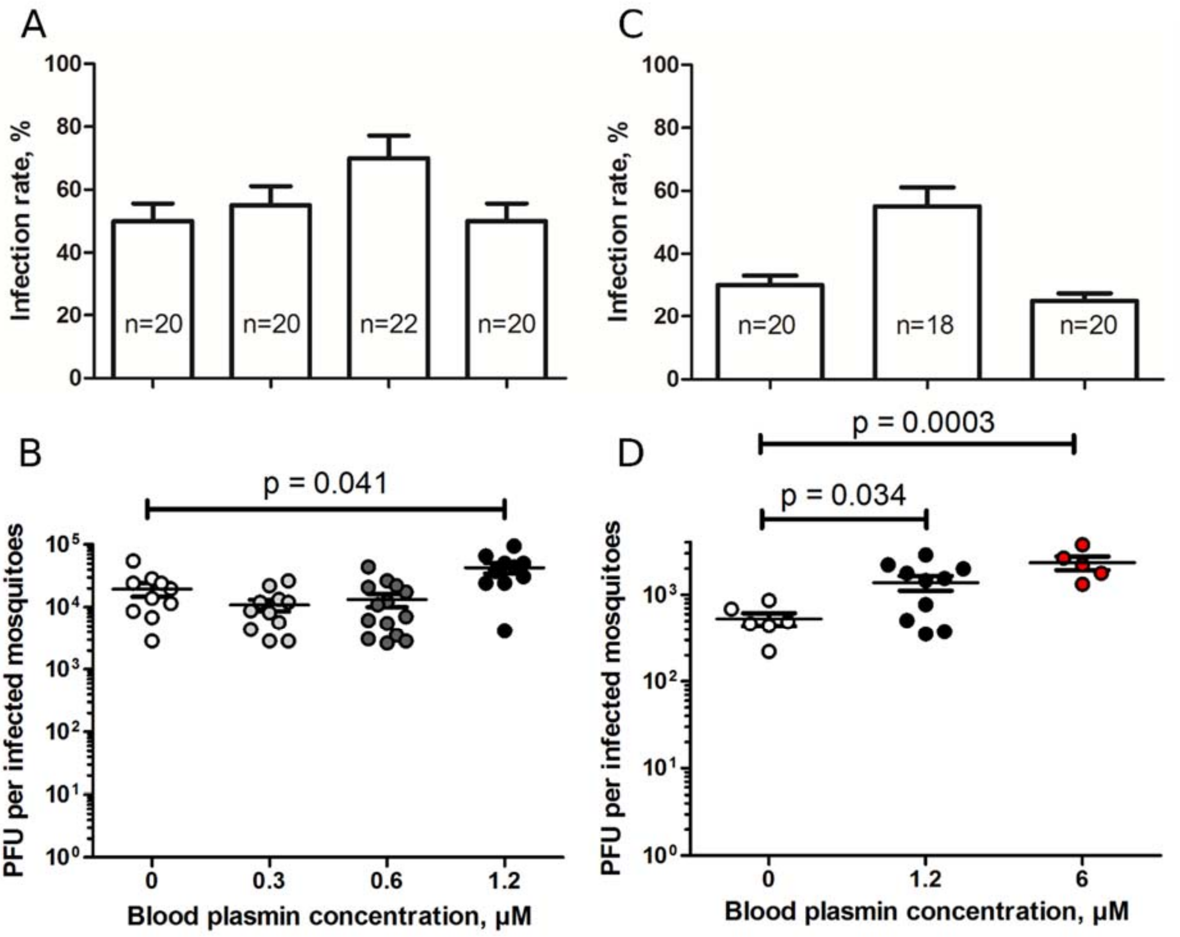

Figure S2. Preliminary dose-response analysis of plasmin impact on mosquito infection, Related to Figure 1. Mosquitoes were offered an infectious blood meal with different concentrations of plasmin. Two independent experiments were conducted with a different stock of DENV, resulting in different intensities of infection. Impact of 0, 0.3, 0.6, 1.2 μM of plasmin on (**A**) infection rate and (**B**) pfu per infected mosquitoes, and impact of 0, 1.2 and 6 μM of plasmin on (**C**) infection rate and (**D**) pfu per infected mosquito. (B and D) Each points represent a mosquito sample, and bars indicate geometric means ± 95% C.I. (A and C) Bars show percentage ± s.e. N, number of mosquito samples. Differences were tested with t-test.

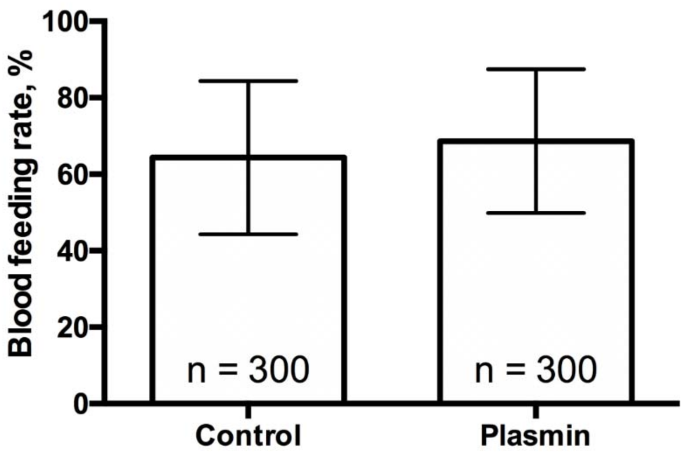

Figure S3. Impact of plasmin supplementation on blood feeding, Related to Figure 1. Mosquitoes were offered either a control or plasmin-supplemented blood meal. Bars show percentage ± s.e. N, number of mosquitoes that were offered the blood meal.

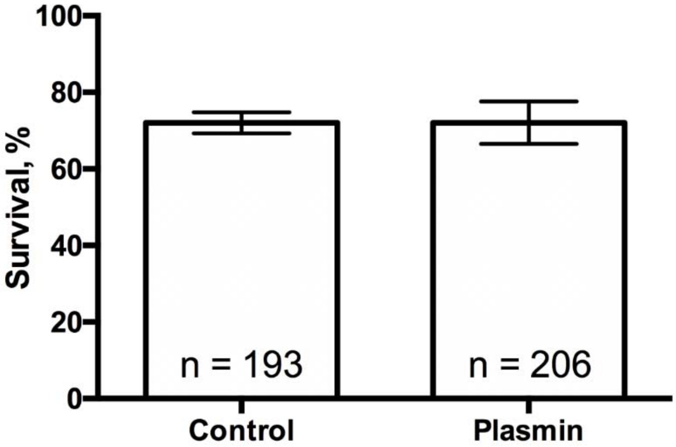

Figure S4. Impact of plasmin supplementation on mosquito survival, Related to Figure 1. Mosquitoes that fed on either control or plasmin-supplemented blood were maintained on sugar solution for seven days before calculating survival. Bars show percentage ± s.e. N, number of blood-fed mosquitoes.

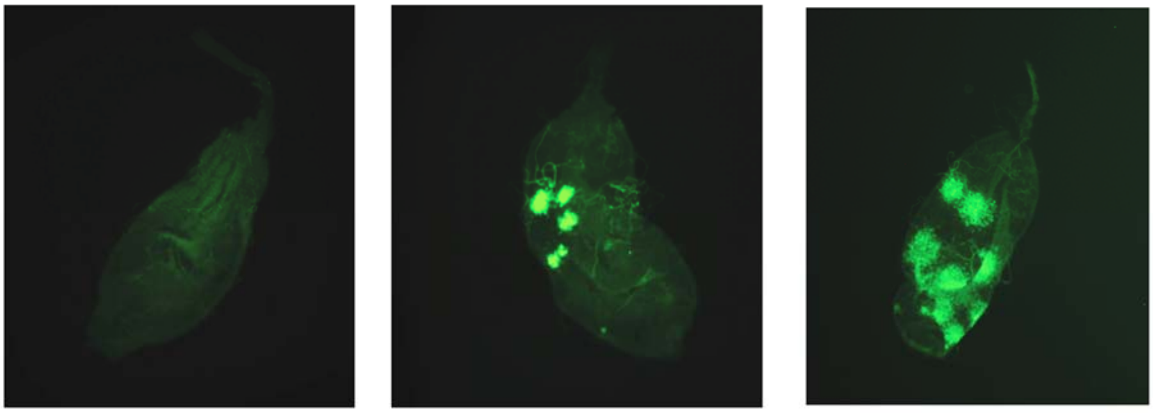

Figure S5. Representative images of non-infected and infected midguts stained for DENV, Related to Figure 1. Midguts were dissected three days post oral infection and stained for DENV envelope. Midguts were visualized with fluorescent microscope.

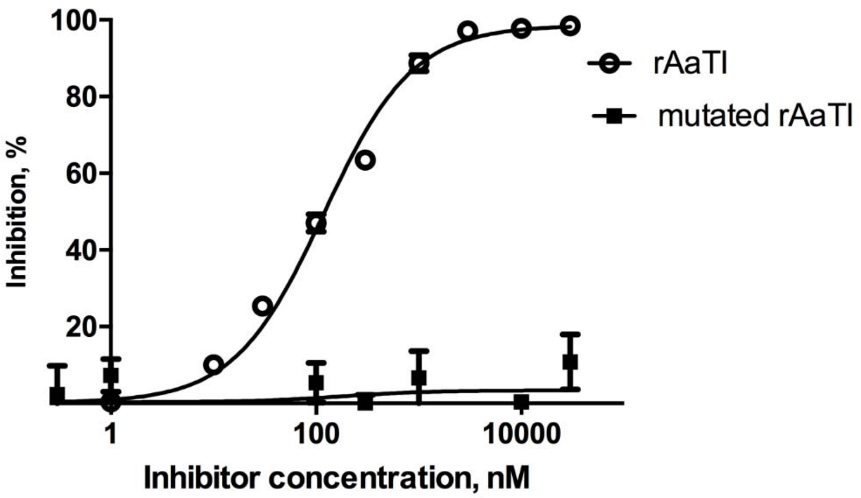

Figure S6. Dose-response curve for rAaTI and mutated rAaTI inhibition of plasmin proteolytic activity, Related to Figures 2 and 4. The curve was used to calculate an IC_{50} of 118 nM. Plasmin proteolytic activity was measured towards S2251 chromogenic substrate and each rAaTI concentration was tested three times. Dots and bars represent mean ± s.e.m.

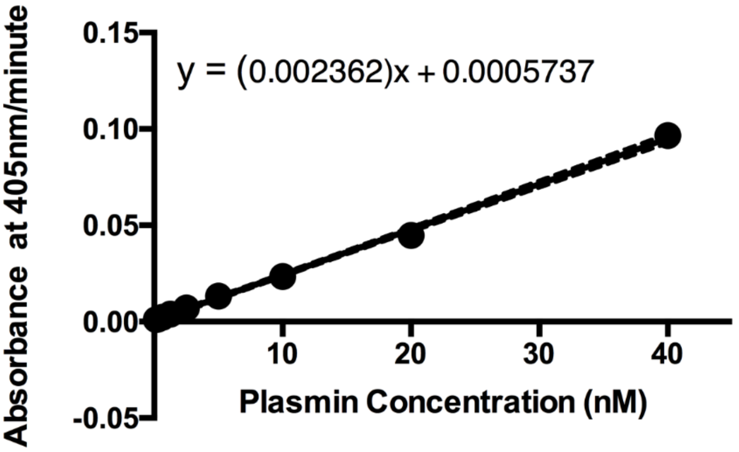

Figure S7. Standard curve for human plasmin proteolytic activity using chromogenic substrate S2251, Related to Figure 3. Fluorescence was measured after incubating S2251 with different concentrations of plasmin (0.078, 0.156, 0.312, 0.625, 1.25, 2.5, 5, 10, 20, 40 nM). Each plasmin concentrations was tested in triplicates and dots represent means. Equation indicates linear equation. Plasmin activity in blood meals was measured using the same method and plasmin concentration was calculated using the linear equation.

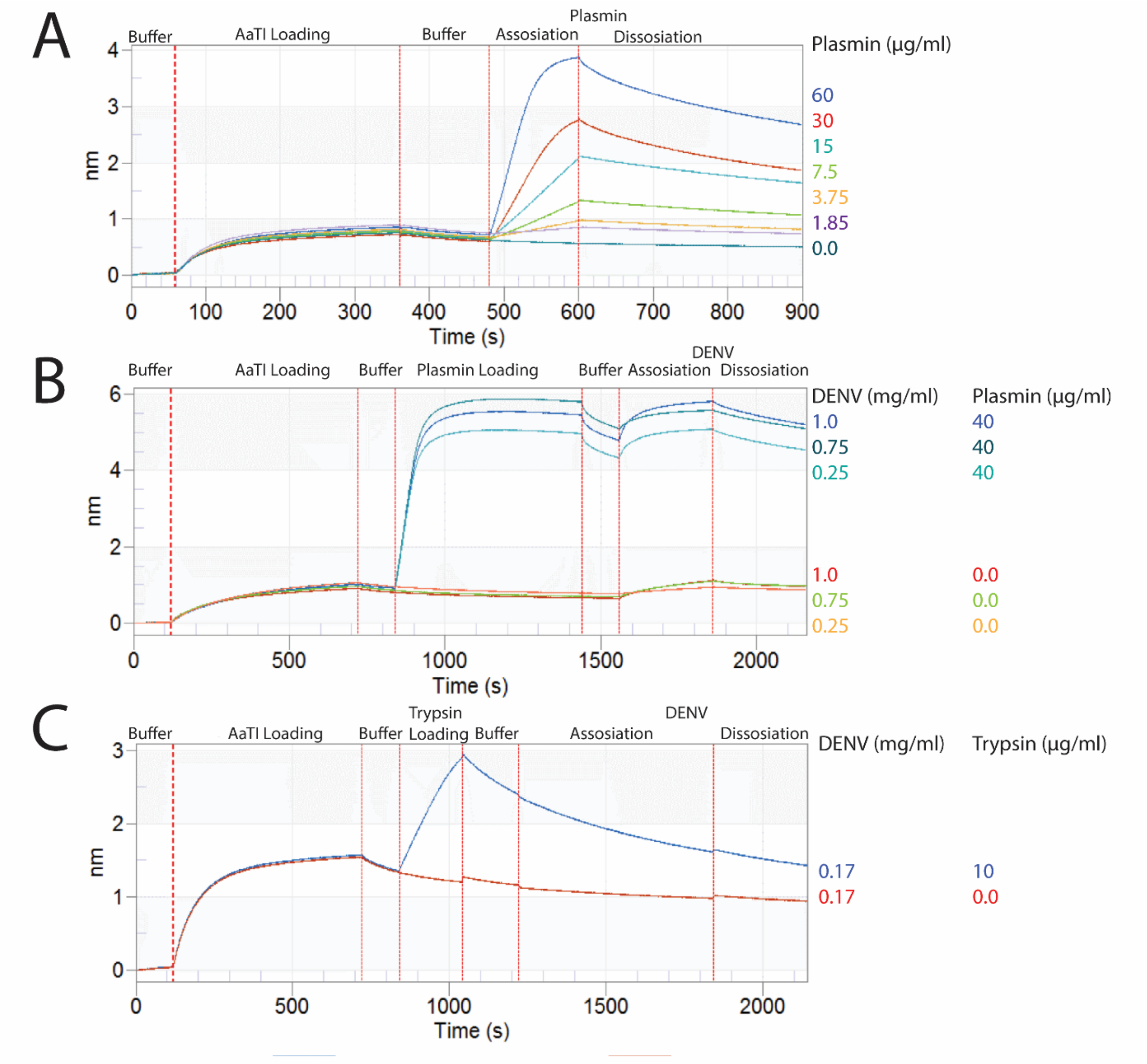

Figure S8. Raw data for BLI experiments, Related to Figure 5. (A) rAaTI-plasmin interaction. (B) rAaTI-plasmin complex interaction with DENV. (C) rAaTI-trysin complex interaction with DENV. Each steps in the BLI experiment is separated by a red dotted line and the composition of the wells are labelled on the top. The concentration of Plasmin/DENV present in the association step is labelled with the respective colours.

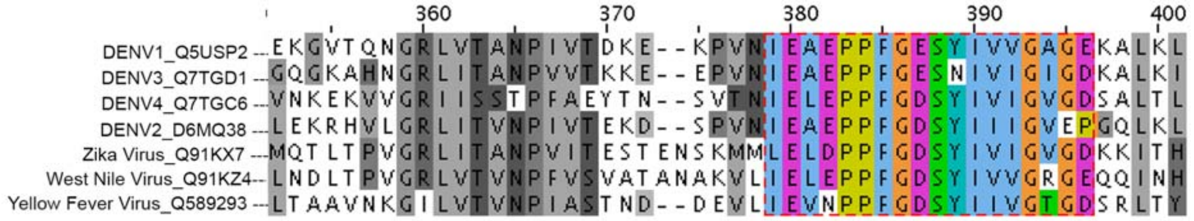

Figure S9. Alignment of E proteins from different flaviviruses, Related to Figure 5. Residues 379 – 396 from representative DENV1, DENV2, DENV3, DENV4, Zika virus, West Nile virus and yellow fever virus are shown. Predicted plasminogen binding sequence [1] is indicated by a red dotted line box.

Supplemental Tables

Table S1. Primer sequences used for dsRNA production and qPCR, Related to figure 2 and 3.

		**Forward primer (5'-3')**	**Reverse primer (5'-3')**
qPCR	*Actin*	GAACACCCAGTCCTGCTGACA	TGCGTCATCTTCTCACGGTTAG
	AaTI	CGGATCTCGGAAGAGCCAAT	GTCACATGCTTGATCGGCAA
dsRNA	*AaTI*	ATGCGCCATATTGGAGTGTT	CTCTTCCGAGATCCGAGTTG
	LacZ	ACACCAACGTGACCTATCCC	CCGCCACATATCCTGATCTT

Table S2. Final concentration of protease and their respective substrates in the reaction mixture, Related to figure 2.

Protease	Concentration, nM	Substrate (Supplier)	Concentration, mM
Plasmin	3.61	S2251 (Chromogenix)	1.2
Factor XIIa	20	S2302 (Chromogenix)	1
Factor XIa	0.125	S2366 (Chromogenix)	1
Factor Xa	0.43	S2765 (Chromogenix)	0.65
Factor IXa	333	FIXa (Spectrozyme)	0.4
Factor VIIa	460	S2288 (Chromogenix)	1
Kallikrein	0.93	S2302 (Chromogenix)	1.1
Alpha Thrombin	0.81	S2238 (Chromogenix)	0.1

Transparent Methods

Mosquito colony and rearing

An *Aedes aegypti* mosquito colony was established in 2010 from eggs collected in Singapore (Mendenhall et al. 2017). Larvae were reared in Milli-Q water and fed with a mixture of liver powder (MP Biomedicals) and fish food (Tetramin Crisps Pro). Adults were maintained in rearing cages (Bioquip) supplemented with 10% sucrose solution and water. The insectary was held at 28°C with 50% humidity on a 12:12h dark:light cycle.

Virus propagation

The DENV2 NGC (New Guinea Colony) strain was propagated in C6/36 *Aedes albopictus* cells (ATCC CRL-1660) cultured in RPMI (Gibco) supplemented with 2% FBS (Research Instruments Pte). Media were harvested five days post-inoculation, aliquoted and stored at -80°C. Viral titer was measured. For Bio-Layer Interferometry experiments, the DENV2 virus was propagated in Serum-Free media.

Plaque assay titration

Titration was conducted from individual mosquitoes homogenized using a bead Mill homogenizer (Mini-Beadbeater, Biospec Products) or artificial blood meals. Samples were filtered through 0.22 µm filter (Sartorius) and plaque forming unit per ml (pfu/ml) was determined from BHK-21 cells (ATCC CCL10) as previously described (Manokaran et al. 2015).

AaTI RNAi depletion

Double-stranded RNA (dsRNA) targeting *AaTI* (AAEL006007) and *LacZ* genes were generated using MEGAscript T7 Kit (Thermo Fisher Scientific) from PCR products amplified with T7-

flanked primers (Table 1). *LacZ* PCR template was produced from a plasmid. DsRNA was purified using E.Z.N.A total RNA kit I (OMEGA Bio-Tek), diluted to 3 µg/ml in DEPC-treated water and annealed by heating to 95°C followed by slow cooling. Sixty-nine µl of dsRNA were injected into thoraces of 2- to 3-day-old mosquitoes anaesthetized on ice, using a glass capillary tube mounted on a Nanoject II injector (Drummond).

AaTI mRNA quantification in midgut

We quantified *AaTI* expression to validate RNAi knock down and to determine AaTI response to blood feeding and infection. For each replicate, ten midguts were dissected and homogenized with a bead Mill homogenizer (Mini-Beadbeater, Biospec Products). Total RNA was extracted using E.Z.N.A total RNA kit I (OMEGA Bio-Tek) and reverse-transcribed using iScript cDNA Synthesis Kit (Bio-Rad). *AaTI* mRNA was quantified by qPCR with SensiFAST SYBR No-ROX kit (Bioline) in a CFX96 Touch Real-time PCR Detection System (Bio-Rad). *Actin* (AAEL011197) mRNA was quantified for normalization. Table S1 shows the primers used for qPCR. Three replicates were conducted per condition.

Oral infection

Two- to three-day-old female mosquitoes or six to eight-day-old dsRNA-injected mosquitoes were sugar-deprived for 24h and given an infectious blood meal containing 600 µl of washed erythrocytes from SPF pig's blood (PWG Genetics), 75 µl of 5 mM ATP (Thermo Scientific), 75 µl of human serum (Sigma) (to compensate for the removed pig blood serum and reconstitute a normal blood composition) and 750 µl of virus diluted in RPMI media (Gibco). To test for plasmin and AaTI impact, the RPMI half of the blood meal was complemented with 75 µl of 2 mg/ml of human plasmin (Athens R & D, USA) and 300 µl of 5 mg/ml recombinantly expressed

AaTI, respectively. Total blood meal volume and inoculum concentration were the same in each condition as plasmin and AaTI volumes replaced extra RPMI used for virus dilution. The viral titer in blood meal was 3×10^6 pfu/ml as validated by titration. Mosquitoes were offered blood meals for two hours using a Hemotek membrane feeder system (Discovery Workshops) covered with a stretched pig intestine membrane. Fully engorged mosquitoes were then maintained in cages supplemented with 10 % sugar solution in an incubation chamber with conditions similar to the insect rearing ones. Whole mosquito titration was conducted after seven days and midgut staining after three days of incubation.

Immunostaining of mosquito midgut

Mosquito midguts were dissected in 1X PBS three days post oral infection. At this time, ingested blood is digested. Midguts were fixed in 4% PFA (Paraformaldehyde) for 1h at room temperature, washed two times in 1X PBS, permeabilized with 0.5 % triton-X 100 in 1X PBS for 1h at room temperature, washed three times with 0.1 % Tween-1X PBS, blocked with the blocking solution (0.1 % Tween20, 1 % horse serum in 1X PBS) for 3h at room temperature, incubated with a 1:2000 anti-dengue envelope mouse antibody (4G2) in blocking solution overnight at 4°C, washed three times with 0.1% Tween-1X PBS, incubated with 1:2000 anti-mouse IgG-Alexa 488 (Axil Scientific) in blocking solution for 3h at room temperature, and washed three times with 0.1% Tween-1X PBS. Midguts were mounted on a glass slide using Prolong-Gold anti-Fade Containing DAPI (Life Technology) and visualized with a fluorescence microscope (Olympus). Number of infected focus per midgut were counted.

Production of recombinant AaTI (rAaTI)

Bacteria-codon optimised *AaTI* coding sequence was obtained from Genscript, cloned into the pet-M plasmid (Fig. S1), transformed into SHuffle® T7 Competent *E. coli* (NEB) cells, expressed at recommended conditions, and purified using a 2-step process: Ni-NTA affinity chromatography followed by gel filtration. After SDS-PAGE validation, the recombinant protein was either used immediately or stored at -80 ^{o}C.

Protease inhibition assay

AaTI inhibitory profiles against 10 serine proteases involved in blood coagulation were examined using a chromogenic assay. Each protease (Hematologic Technologies Inc.) was incubated with rAaTI before the respective chromogenic substrate (Chromogenix) was added. Substrate chromophore release reflected proteolysis activity and was quantified using infinite m200 plate reader (Tecan) at 405 nM. Total reaction volume was 75 µl and contained 30 µM rAaTI. The final reaction concentrations of the proteases and substrates are detailed in Table S2.

Bio-Layer Interferometry

Biolayer Interferometry (FortéBIO Octet RED 96 instrument, PALL Corporation) was used to study the interactions between DENV, plasmin and AaTI. Plasmin, trypsin and rAaTI were diluted in 50 mM Tris pH7.5, 100 mM NaCl, 0.1 % BSA buffer at room temperature. DENV in serum-free RPMI media was buffer-exchanged with 100 kd cut-off centricon (Millipore) and virus concentration was determined based on the equivalent of DENV envelope protein.

To characterize plasmin-AaTI interaction, Ni-NTA biosensors were pre-loaded with recombinant His-tagged AaTI (40 µg/ml) for 300 s, immersed in human plasmin at different

concentrations (0, 1.85, 3.75, 7.5, 15.0, 30μg/ml) for 120 s followed by equilibration step in the assay buffer for 300 s. Buffer values were subtracted to account for background.

To characterize DENV-plasmin interaction, biosensors preloaded with rAaTI (40 μg/ml) for 600 s were immersed in a plasmin solution (40 μg/ml) for 600 s, equilibrated in the assay buffer for 120 s, immersed in DENV at 0.25, 0.75, and 1.0 mg/ml for 300 s and followed by a dissociation step for 300 s. Additionally, biosensors preloaded with rAaTI only (immersed in plasmin buffer) were immersed in the DENV solutions and used to control for background for each respective DENV concentrations.

To test interactions between trypsin, AaTI and DENV, we diluted bovine trypsin (Sigma) in 50 mM Tris pH7.5, 100 mM NaCl, 0.1 % BSA buffer. Similar to testing for plasmin interaction, biosensors were preloaded with rAaTI and immersed in 10 µg/ml trypsin solution for 200 s followed by equilibration for 120s. A low concentration of trypsin was used to prevent trypsin cleavage of His-tagged rAaTI. The association of rAaTI-trypsin biosensors with DENV at 0.17 mg/ml was allowed for 620 s, and followed by dissociation step for 300 s. DENV-rAaTI interaction values were subtracted as background.

The Octet software, v 9.0.0.14 (PALL) was used to analyse the binding data. Nonlinear regression fitting using 1:1 binding mode provided the best fit and was used to derive the dissociation constants.

Midgut internalization assay

Two- to five-day-old female mosquitoes were sugar-deprived for 24h and offered a blood meal consisting of 40 % volume of washed erythrocytes from SPF pig's blood, 5 % of 100 mM ATP, 5 % human serum, 10 % of 60 mg/ml 70 kDa fluorescein isothiocyanate (FITC)-Dextran (Sigma) and 40 % volume of RPMI supplemented with either: (i) 75 µl of 2 mg/ml human

plasmin, (ii) 10^7 pfu/ml of DENV, (iii) 75 µl of 2 mg/ml human plasmin and 10^7 pfu/ml of DENV, or (iv) 75 µl of 2 mg/ml human plasmin, 10^7 pfu/ml of DENV and 300 µl of 5 mg/ml rAaTI. Mosquitoes were fed for 1 h. Fully engorged females were maintained in conditions similar to those in the rearing room with 10 % sucrose. At 18h post oral feeding, the midguts were dissected, washed three times in PBS to remove dextran that was not inside midgut tissues, and mounted using a DAPI-containing glue (Fluoroshield, Sigma). Pictures were taken with the Eclipse 80i fluorescent microscope (Nikon) and the LSM710 confocal microscope (Zeiss).

Statistical Analysis

Infection level was measured by pfu and infection foci only from either infected mosquitoes or infected midguts. Infection rate was calculated as the number of infected sample divided by the total number of samples. Infection level and infection rate were quantified from the same set of mosquito samples under various conditions. Variations in plaque forming units and foci of infection were compared using multiple t-test after log-transformation. Fisher's exact test was used to compare the percentages of infection. All analyses were done using GraphPad's online QuickCalcs tool.

Supplemental References

Manokaran, Gayathri, Esteban Finol, Chunling Wang, Jayantha Gunaratne, Justin Bahl, Eugenia Z Ong, Hwee Cheng Tan, October M Sessions, Alex M Ward, and Duane J Gubler. 2015. 'Dengue subgenomic RNA binds TRIM25 to inhibit interferon expression for epidemiological fitness', *Science*, 350: 217-21.

Mendenhall, Ian H., Menchie Manuel, Mahesh Moorthy, Theodore T. M. Lee, Dolyce H. W. Low, Dorothée Missé, Duane J. Gubler, Brett R. Ellis, Eng Eong Ooi, and Julien Pompon. 2017. 'Peridomestic Aedes malayensis and Aedes albopictus are capable vectors of arboviruses in cities', *PLoS Neglected Tropical Diseases*, 11: e0005667.

Monroy, V, and BH Ruiz. 2000. 'Participation of the dengue virus in the fibrinolytic process', *Virus Genes*, 21: 197-208.
